# Comparison of the welfare of beef cattle in housed and grazing systems: hormones, health, and behaviour

**DOI:** 10.1017/S0021859623000357

**Published:** 2023-06-29

**Authors:** A. S. Cooke, S. Mullan, C. Morten, J. Hockenhull, P. Le Grice, K. Le Cocq, M. R. F. Lee, L. M. Cardenas, M. J. Rivero

**Affiliations:** 1 School of Life Sciences, College of Science, University of Lincoln, Lincoln, UK; 2 Net Zero and Resilient Farming, Rothamsted Research, Okehampton, UK; 3 UCD School of Veterinary Medicine, University College Dublin, Dublin, Ireland; 4 Bristol Veterinary School, University of Bristol, Bristol, UK; 5 School of Sustainable Food and Farming, Harper Adams University, Edgmond, UK

**Keywords:** animal welfare, animal health, livestock, farming, agriculture

## Abstract

Animal welfare encompasses all aspects of an animal’s life and the interactions between animals. Consequently, welfare must be measured across a variety of factors that consider aspects such as health, behaviour, and mental state. Decisions regarding housing and grazing are central to farm management. In this study, two beef cattle systems and their herds were compared from weaning to slaughter across numerous indicators. One herd (“HH”) were continuously housed, the other (“HG”) were housed only during winter. Inspections of animals were conducted to assess body condition, cleanliness, diarrhoea, hairlessness, nasal discharge, and ocular discharge. Hair and nasal mucus samples were taken for quantification of cortisol and serotonin. Qualitative behaviour assessments (QBA) were also conducted and performance monitored. Physical health indicators were similar between herds with the exception of nasal discharge which was more prevalent in HH (*P* < 0.001). During winter, QBA yielded differences between herds over PC1 (arousal) (*P* = 0.032), but not PC2 (mood) (*P* = 0.139). Through summer, there was a strong difference across both PC1 (*P* < 0.001) and PC2 (*P* = 0.002), with HG exhibiting more positive behaviour. A difference was found in hair cortisol levels, with the greatest concentrations observed in HG (*P* = 0.011), however such a pattern was not seen for nasal mucus cortisol, or for serotonin. Overall, providing summer grazing (HG) appeared to afford welfare benefits to the cattle as shown with more positive QBA assessments, but also slightly better health indicators, notwithstanding the higher levels of cortisol in that group.

## Introduction

Animal welfare is an important component of decision making on farms and is something desired by consumers and wider society (Rivero and Lee, 2022). Within beef systems, one of the most critical decisions to be made is the extent to which cattle are housed or grazing on pasture. Across England, approximately 0.87 of farms use a mixture of housing and grazing and 0.04 exclusively house their cattle (DEFRA, 2019). There are potential welfare benefits to both housing and grazing, as well as environmental, economic, and social benefits. Housing protects animals from adverse weather and ground conditions that could otherwise cause welfare losses such as lameness (Gregory *et al*., 2006), though properly managed grazing has also been found to reduce similar welfare losses (Haskell *et al*., 2006). There is growing evidence which suggests cattle find grazing environments more psychologically ‘rewarding’ than indoor environments (Crump *et al*., 2021) and that access to pasture can provide welfare benefits (Charlton and Rutter, 2017; de Graaf *et al*., 2017). Inevitably, decisions surrounding housing/grazing will impact diet which will in turn impact performance. This is reflective of the fact that housed systems are typically used as a means to intensify production.

The demand for ‘ethical’ animal products is increasing, driven by a moral view from consumers about the societal responsibility to care for animals (Frey and Pirscher, 2018). Consumers generally view pasture-reared livestock to be more ethical on the basis of animal welfare and environmental standards (Stampa *et al*., 2020; Wilkinson *et al*., 2020; Xue *et al*., 2010). This also provides an economic opportunity due to the price premiums available for animal products perceived to be of higher welfare standards (Cornish *et al*., 2020; Lagerkvist and Hess, 2011; Yang and Renwick, 2019).

Welfare work on cattle has overwhelmingly focussed on the dairy sector, due to larger herd sizes, greater preference towards continual housing (relative to beef) and the fact that the output (milk) is produced continuously and thus any short-term adversity has an instant financial impact. There is, therefore, a need to improve our holistic understanding of beef cattle welfare. Assessing animal welfare, however, is challenging. Welfare is not one single variable; it is a complex interaction of all aspects of an animal’s existence and being. It is therefore essential to address welfare assessment holistically, considering factors spanning different aspects of the animal (Mellor and Reid, 1994; Rivero and Lee, 2022).

Of the five ‘Domains’ of animal welfare, i.e., Nutrition, Environment, Health, Behaviour and Mental State (Mellor, 2016), the health domain of animal welfare is perhaps the most apparent indicator, being evidenced by the animals physical health and condition, factors that are often easily visible, unambiguous, or part of wider husbandry checks and veterinary care. These can be assessed by looking into the veterinary records of animals or by direct assessment, using procedures such as those outlined by the Welfare Quality protocol (Welfare Quality®, 2009) and the Beef Cattle Assessment Protocol (AssureWel, 2017), both of which cover health indicators such as: discharges, hairlessness, and lameness. Behavioural assessment, on the other hand, is an element that can provide insights into the behavioural domain of animal welfare. In recent years, Qualitative Behaviour Assessment (QBA) (Wemelsfelder, 2008) has become a popular approach both academically and commercially (Battini *et al*., 2018; Ceballos *et al*., 2021; Cooke *et al*., 2022; Fleming *et al*., 2016; Waitrose & Partners, 2021). The method has also shown potential in the detection of illness (Health ‘domain’), such as mastitis (de Boyer des Roches *et al*., 2018), though studies investigating correlations with other health, clinical, and welfare indicators have yielded mixed results (Battini *et al*., 2018; Brscic *et al*., 2009). However, QBA should not be used in isolation and may have limited correlation with other welfare assessments such as the Welfare Quality protocol (Andreasen *et al*., 2013). Hormone levels can act as indicators and drivers of both health and mental state (one of the domains of animal welfare). For example, cortisol is a steroid hormone that is widely regarded as an indicator of stress and anxiety in cattle (Bristow and Holmes, 2007; Mitchell *et al*., 1988) and can be elevated in response to events such as social separation (Boissy and Le Neindre, 1997) but also overcrowding (Friend *et al*., 1977). However, whilst there has been some assumption that cortisol is indicative of cattle welfare, van Eerdenburg *et al*. (2021) found that it did not correlate with other welfare assessments such as the Welfare Quality protocol. Serotonin is an important neurotransmitter which regulates a variety of bodily processes including the secretion of growth hormones (Kasuya *et al*., 2010),regulation of lactation (Collier *et al*., 2012), and the immune system (Wu *et al*., 2019). In humans, low levels and deficiencies of serotonin have been linked to depression and various mood disorders (Cowen and Browning, 2015). Sunlight has also been found to promote the turnover and synthesis of serotonin in humans (Lambert *et al*., 2002), though this has not been studied in cattle, thus there is the potential that restricting exposure of cattle to sunlight, due to housing, could influence serotonin concentrations. Non-invasive hormone quantification is possible using saliva or nasal mucus, which have been found to correlate with serum concentrations (Houbeau *et al*., 1986) and are matrices widely used to quantify various biomarkers (Johnsen *et al*., 2019; Silva *et al*., 2012). The environmental domain can interact strongly with the other four domains. For beef cattle, two of their most common environments are open pasture or enclosed barns, which are largely different environments in terms of factors such as space and climate. This ties in closely to the nutritional domain as housed and grazed cattle differ greatly in the feeds that they received, but also how they consume them.

This work aimed to address the question: How do the health and welfare of beef cattle differ between housed systems and conventional systems? For that purpose, we assessed two beef cattle herds, with and without access to pasture after winter housing, across a variety of health, welfare, and performance metrics.

## Materials and methods

This study compared the welfare of two herds of finishing beef cattle, one in a housed system and the other in a conventional system. Welfare and health were assessed across four key areas:

1. Physical health – Body condition and health inspections conducted on an individual animal basis. Also, veterinary records for any treatments.
2. Behavioural – QBA was conducted at a group level.
3. Endocrinology – Concentrations of cortisol and serotonin in both hair and nasal mucus on an individual animal basis.
4. Performance – Analysis of monthly liveweight data.

### Site and population description

The experiment was conducted at the Biotechnology and Biological Sciences Research Council's National Bioscience Research Infrastructure, hosted at Rothamsted Research: The North Wyke Farm Platform (Devon, UK) (Orr *et al*., 2019, 2016; Takahashi *et al*., 2018). Weather patterns for the study period are included in Supplement A.

Two beef rearing systems were compared. The first system had cattle housed all year around, named “**HH**”. The second system saw cattle housed during the winter months, but grazing on pasture the rest of the year, named “**HG**”. Both herds had 30 head of the breeds stabiliser (ST) and stabiliser cross (STX) cattle, all of which derived from the same suckler herd. Animals were allocated from the suckler herd at weaning (27 October 2020) to one of the two systems. At allocation both resultant herds had the same mean age and weight (235 days [s.d. 18.2], 332 kg [s.d. 36.4]). The sex ratio was similar between herds (F:M, 21:9 and 23:7, respectively) as was breed ratio (ST:STX, 24:6 and 23:7, respectively).

**HH** (housed-housed)– The herd was housed from weaning (27 October 2020) until finishing at ~14-15 months age. During this time, they received grass (predominately perennial ryegrass; *Lolium perenne*) silage *ad libitum*, 4 kg per head of rolled barley (*Hordeum vulgare*), and 0.3 kg per head of wheat (*Triticum aestivum*) distiller’s grain.

**HG** (housed-grazing) – This herd were housed from weaning (27 October 2020) through winter, during which they received grass silage (predominately perennial ryegrass) *ad libitum*. In spring, the herd were turned out onto pasture (13April 2021), which was dominated (>0.60) by perennial ryegrass, with a smaller abundance of creeping bent (*Agrostis stolonifera*), Yorkshire fog (*Holcus lanatus*) and marsh foxtail (*Alopecurus geniculatus*) also contributing to the sward; legumes comprised <0.01 of the overall composition (Orr *et al*., 2019, 2016).

Both herds were housed in separate, but adjacent, straw-bedded barns built to the same specification/blueprints. The available space was 24 x 11 m which included a bedding area (7.5 x 24 m) and concrete floored area (3.5 x 24 m). Straw bedding was added to pens daily using a tractor trailed straw chopper. The concreted area was scraped every morning to remove dung and urine using a tractor mounted yard scraper. The entire daily feed ration was distributed along the length of a ground-level feed passage using a tractor trailed forage mixer wagon every morning after bedding and scraping. The feed passage was adjacent to the concreted area and allowed access to feed through diagonal bars. Every evening the remaining feed was pushed up using a tractor mounted silage pusher to ensure it was able to be reached by the animals. The forage mixer wagon was equipped with weigh cells enabling accurate measurement of rations.

Animals were weighed every ~4 weeks and selected for slaughter on the basis of visual assessment and palpation of skeletal reference points as they approach target weights (approx. 620-680 kg for steers, 550-580kg for heifers) and fat class (4L on the union scale: – moderately high fat cover, lean). Throughout the course of the study, the number of cattle in the HH herd decreased as they were sold to slaughter as part of normal management practice (Supplement B) this was due to the high growth rates compared to the HG herd.

### Physical assessments

Cattle were examined across eight health indicators that covered a range of symptoms associated with common disease/infections of cattle. These were selected and adapted from the Welfare Quality Protocol (Welfare Quality, 2009) and AssureWel protocol (AssureWel, 2017) (Table 1). Checks were conducted along the right side of their bodies whilst they were in the race, waiting to be weighed, thus mitigating for potential time and stress of assessments. Two assessments were conducted during the winter (2 November 2020 and 16 November 2021), when both herds were housed, and two conducted in the summer (16 May 2021 and 21June 2021), when the HH herd were housed but the HG herd were on pasture. Body condition scoring (BCS) was conducted at the same time, scores were given in line with NADIS guidelines (NADIS, 2010), to whole integers (1-5) (Table 1).

### Qualitative Behaviour Assessment

Qualitative behaviour assessment (QBA) was conducted on a weekly basis for both herds. Assessments were conducted by a single assessor with previous experience conducting QBA on beef cattle and who is a qualified professional in animal science with approximately seven years’ experience. In previous studies (Cooke *et al*., 2022) the assessor showed a very high level of inter-observer reliability (0.78-0.87) with other assessors. Assessments were conducted during daylight hours and both herds were observed within a 30-minute window on any given day, excluding the short windows during which husbandry activities (bedding, cleaning, feed being added) were actively occurring. Cattle were observed for 10 minutes in their ‘home’ environment (e.g., housing or pasture). The assessor watched, stationary, from the outside of the feeding passage of field boundary. If it was felt that the animal’s behaviour changed due to their presence the assessor was instructed to wait two minutes for that effect to subside – though this was never necessary as the cattle were familiar with the presence of people. Cattle were scored on a 125 mm visual analogue scale across 20 terms, based on the prevalence and intensity of the relevant factor. Those terms were: Active, Agitated, Apathetic, Bored, Calm, Content, Distressed, Fearful, Friendly, Frustrated, Happy, Indifferent, Inquisitive, Irritable, Lively, Playful, Positively occupied, Relaxed, Sociable, Uneasy (terms taken from the Welfare Quality protocol, see Supplement C for brief descriptions of these terms).

### Hormone levels

Cortisol and serotonin were quantified in the hair and nasal mucus of the cattle. Cortisol was considered as an indicator of stress and anxiety (Boissy and Le Neindre, 1997; Bristow and Holmes, 2007) with nasal concentrations being correlative to those in plasma and saliva (Houbeau *et al*., 1986). Serotonin was considered in relation to its role as a neurotransmitter influencing mood (Marrero *et al*., 2019) with higher levels assumed as preferential.

## Sample collection and preparation

Hair and nasal mucus samples were taken at the same time as physical checks were conducted. Hair was taken using an electric shearer, from an unshaven/unclipped area around the base of the neck and between shoulder blades. Hair length and growth rates were not measured, though no difference was apparent between herds. Hair samples were stored at -20ºC prior to further preparation. Samples were prepared by protocol adapted from van Eerdenburg *et al*. (2021) and Sharma *et al*. (2019). Briefly: hair was washed, dried, and ground. Methanol was then used to extract hormones from the ground hair, the methanol was evaporated off and the remanent material suspended in PBS (phosphate-buffered saline) for analysis. Nasal mucus was collected using sterile cotton swabs, to a nostril depth of approximately 5 cm. Methanol extraction was conducted on the mucus, methanol evaporated off, and the material suspended in PBS for analysis. More information on hormone extraction is included in Supplement D.

Hair and nasal mucus extracts (suspended in PBS) were analysed for cortisol and serotonin concentrations. Cortisol was quantified by enzyme linked immunosorbent assay (ELISA) using a commercially available kit (Salimetrics, USA, #1-3002). Methodology was adapted from the manufacturer instructions and are further detailed in Supplement E. Serotonin was also quantified by ELISA using a high sensitivity kit (DLD Diagnositka GMBH, Germany, EA 630/96) in line with manufacturer instructions (see Supplement E). Once concentrations in the sample extracts were quantified, concentrations relative to the amount of material (hair or nasal mucus) were calculated.

### Data analysis

Results for physical health indicators were analysed using cumulative link models (CLM). The dependent variable was the score for the relevant indicator and the independent variables were season, timepoint (nested within season) and herd. The exception to this was the measure of ‘hairlessness’ which had insufficient variance of results for CLM analysis, instead, a general mixed effect model (GLMM) was conducted.

Results from QBA were subject to PCA analysis with scaling and centring (variables centred to a mean of 0 and scaled to a standard deviation of 1). From this, first and second principal components (PC1 and PC2) were derived. Results were reported with 95% confidence ellipses for each treatment each season. For each season, two-tailed *t*-tests were conducted to compare herds across both PC1 and PC2.

Hormone data was analysed using repeated measures ANOVAs with concentrations as the dependent variable and timepoint and herd as independent variables. Post-hoc Tukey testing was then conducted to determine differences between herds at all timepoints.

Cattle growth rates were compared using a general linear model (Gamma distribution) that considered herd, breed, and sex as factors.

Across all timepoints and both herds, hormone concentrations, negative health scores, and performance (average daily gain) were compared against each other by way of Kendall’s rank correlation. Negative health scores were determined per animal, per timepoint, as the sum of scores for cleanliness, diarrhoea, hairlessness, nasal discharge, ocular discharge, swelling, lesions, and lameness.

Statistical analysis and figures were performed in R (i386 4.1.2) (R Core Team, 2021) and R Studio (2021.09.1) (R Studio Team, 2020) using packages ggplot2 (Wickham, 2016), ggfortify (Tang *et al*., 2016), ordinal (Christensen, 2019), corrplot (Wei and Simko, 2021), and Cairo (Urbanek and Horner, 2020).

## Results

### Physical health indicators

**Fig. 1:**
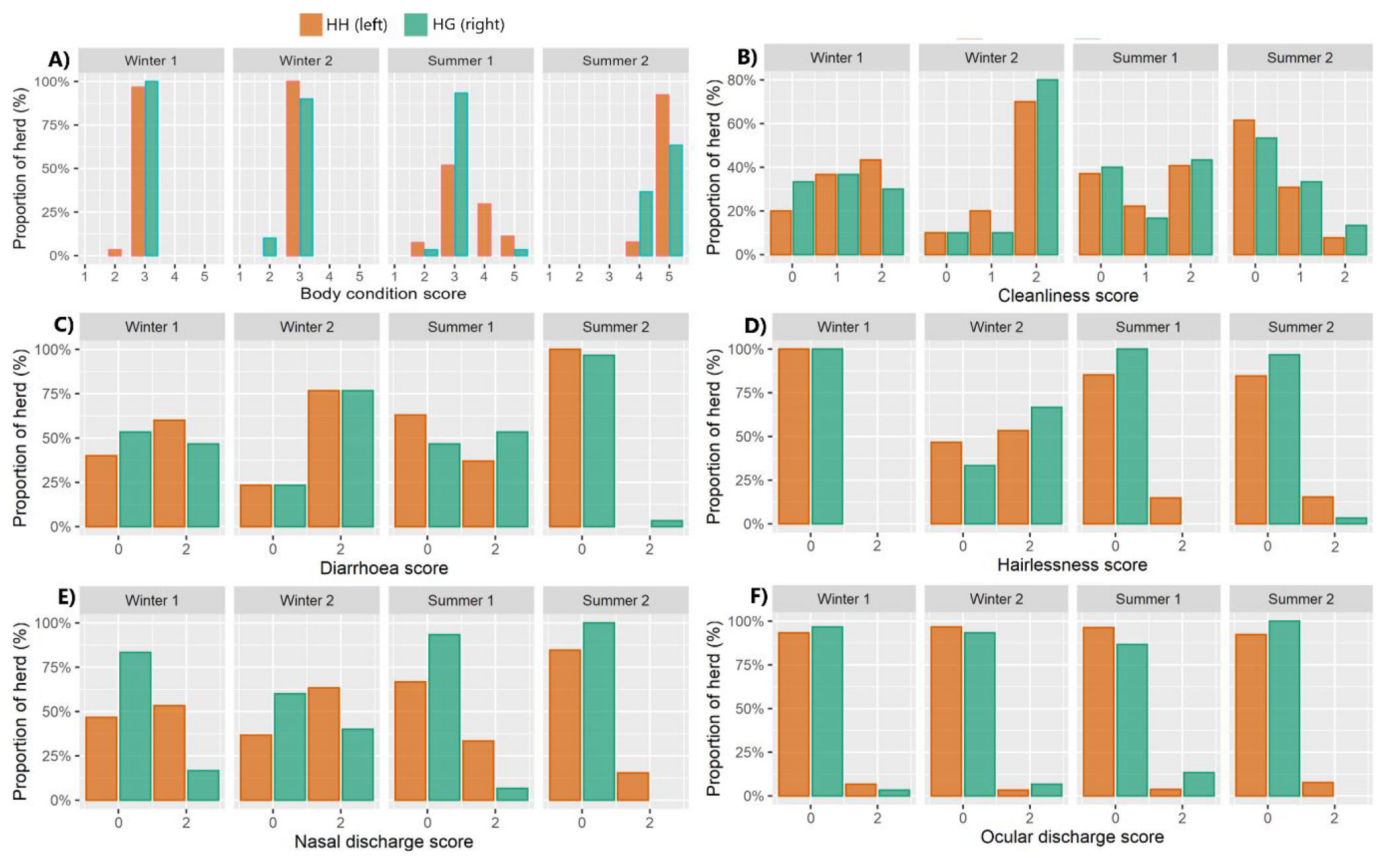
Results of physical inspection of animals for the purpose of scoring physical health indicators. Results are separated between herds and across four time points. (a) body condition scores (b) cleanliness scores (c) diarrhoea scores (d) hairlessness scores (e) nasal discharge scores (f) ocular discharge scores. Results for lameness, swelling and lesions are described in the text.

Differences were observed in scores for physical health indicators both between herds and across time (Figure 1). **Lameness:** no incidences were observed over the study period. **Swelling:** one swelling was observed on the head/neck of an animal in the HH herd at timepoint Summer 1, which was scored as 2 (≥ 2cm diameter). **Lesions:** one lesion was observed on the head/neck of an animal in the HH herd at timepoint Summer 1. **BCS:** scores were significantly greater in the HH herd than HG (*z* = 3.367, *P* < 0.001). During winter, across both herds scores remained relatively stable (*z* = 0.67, *P* = 0.503). Scores were significantly higher in summer than in winter in both herds (*z* = 7.90, *P* < 0.001) and increased significantly over summer (*z =* 7.19, *P* < 0.001). **Cleanliness:** no significant difference was found between the two herds across the study period (*z* = 0.03, *P* = 0.910). There was a significant difference in cleanliness scores across time (*z* = 3.95, *P* < 0.001) and, notably, during winter cleanliness scores were far worse at the end compared to beginning. Cleanliness appeared to improve over summer with animals significantly cleaner at the end compared to start (*z* = -2.68, *P* = 0.007) and, overall, animals were cleaner in the summer than in the winter (*z* = -3.10, *P* = 0.002). **Diarrhoea:** scores followed a similar trend to cleanliness scores. Higher (worse) scores were seen in winter compared to summer (*z* = 3.711, *P* < 0.001), scores were worst at the end of winter compared to start (*z* = 2.64, *P* = 0.008) and there was an improvement over summer (*z* = -3.41, *P* < 0.001). At timepoint Summer 2, the diarrhoea rate (at score 2) was higher for the HG herd (43.3%) compared to the HH herd (25.9%). Across the entire study period there was no statistically significant difference between the two herds (*z* = 0.19, *P* = 0.951). **Hairlessness:** incidences were relatively uncommon, though there was a peak for both herds at winter 2 which was significantly greater than at other time points (*z* = 5.14, *P* < 0.001). There was no significant difference between herds (*z* = 0.53, *P* = 0.597). **Nasal discharge:** At all timepoints scores were significantly (*z* = 4.38, *P* < 0.001) higher (worse) for the HH herd than for HG. A similar temporal effect to cleanliness and diarrhoea was seen with scores being greatest in winter (*z* = 2.96, *P* = 0.004) with incidence increasing (albeit not significantly at α = 0.05) over winter (*z* = 1.95, *P* = 0.051) and decreasing through summer (*z* = 1.72, *P* = 0.086). **Ocular discharge:** incidences were uncommon in both groups and thus there was no significant difference between herds (*z* = 0.40, *P* = 0.687) or seasons (*z* = 0.713, *P* = 0.476).

Veterinary treatment records showed that a total of six different conditions were treated for during the study (Table 2), three of those were observed in the HH herd and five in the HG herd. The total number of non-routine veterinary treatments was greater (18) in the HG herd compared to HH (5) during the winter, however these were predominantly preventative measures. In the summer, there were four bouts of mange treated for in the HH herd, compared to none in the HG herd, whilst five other treatments occurred in the HG herd.

### Qualitative Behaviour Assessment

**Fig. 2:**
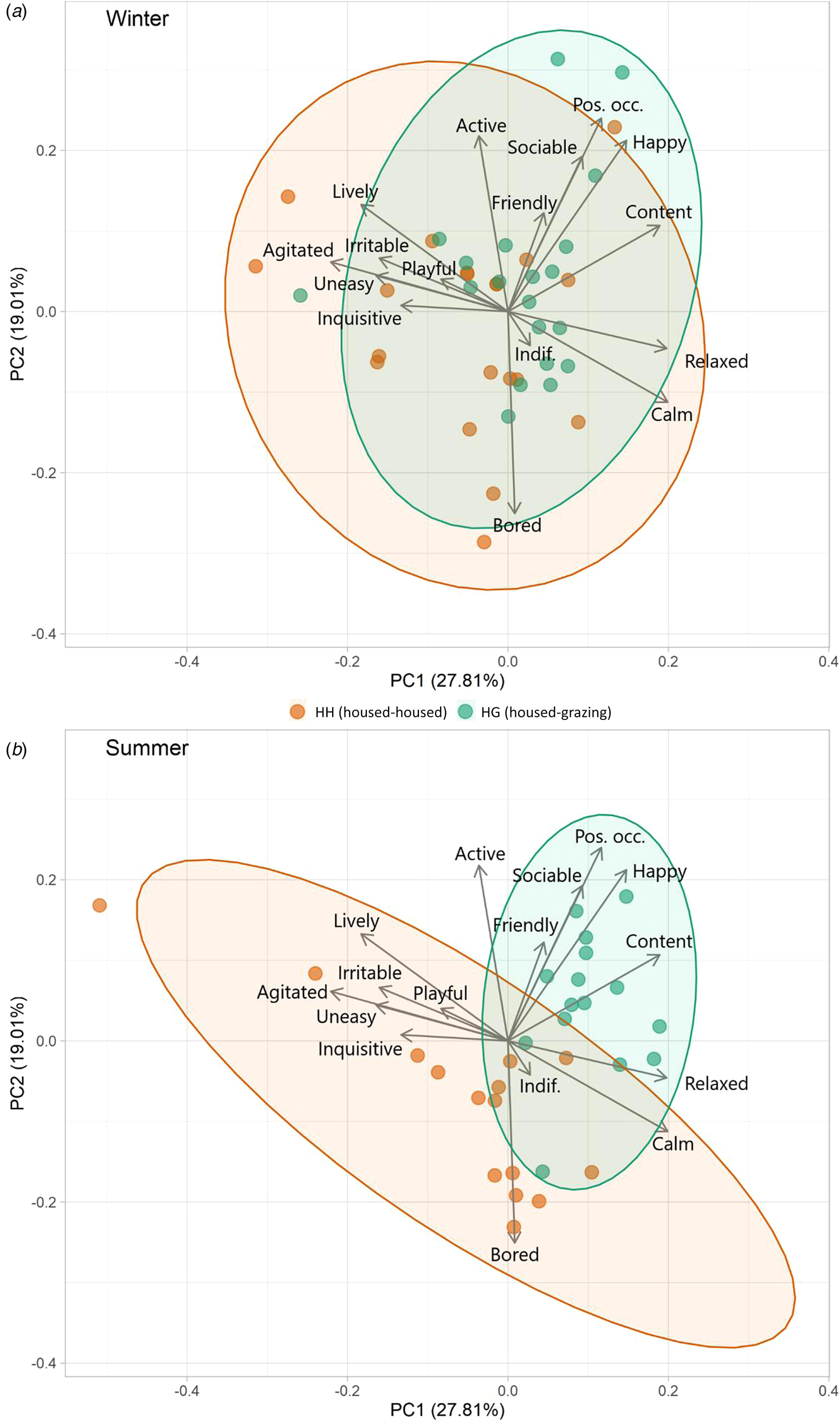
Biplots of PCA results from winter (a) and summer (b) QBA results, showing distribution of data for each herd. Ellipses represent 95% confidence.

PC1 and PC2 accounted for 0.49 of variance (Table 3 and Figure 2).PC1 was defined as ‘arousal’ due to the high loadings for “Calm”, “Relaxed”, “Agitated” and “Lively”, whilst : arousal and PC2 was defined as “mood” based on the high loadings for “Bored” and in line with the Welfare Quality® protocol. During winter, the herds differed significantly across PC1 (*t* = 2.23, *P* = 0.032) but not PC2 (*t* = 1.51, *P* = 0.139). The difference in arousal is represented by marginally calmer outcomes observed for HH relative to HG. During summer there were strong and significant differences between herds across both PC1 (*t* = 3.78, *P* < 0.001) and PC2 (*t* = 3.49, *P* = 0.002). Outcomes of HG were associated strongly with constructive and socially cohesive behaviours. There was larger variation in behaviours for HH, but with an inclination towards negative behaviours and boredom.

### Hormone levels

**Fig. 3:**
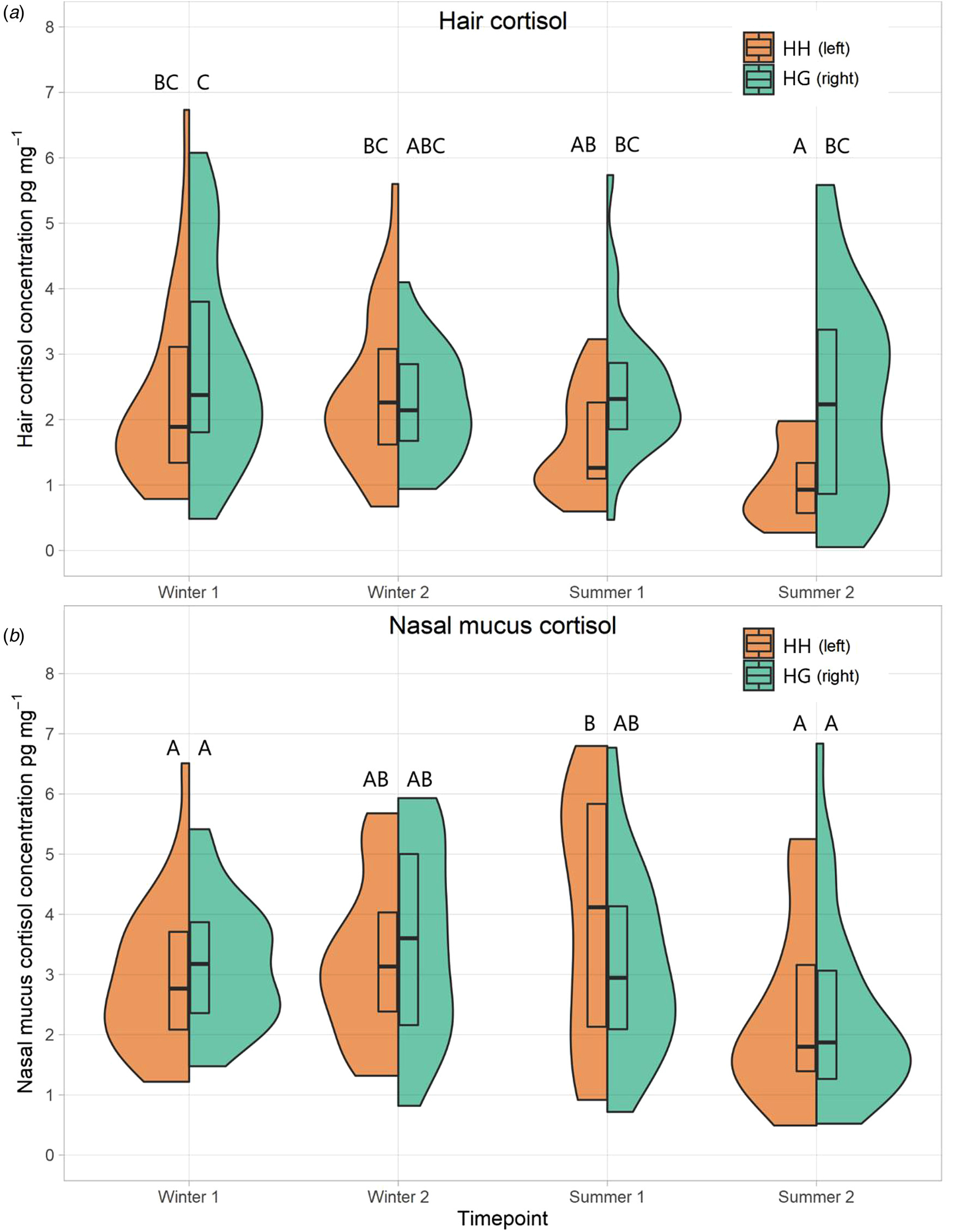
Split violin plots of cortisol concentrations (pg mg^-1^) for both herds across all time points in both hair (a) and nasal mucus (b). The left half of each plot is the HH herd (coloured in HH), and the right half is the HG herd (coloured in HG). Boxes in the middle represent Q1, median and Q3. Plots that do not share a letter are significantly different to one another.

For the HG herd, hair cortisol concentrations (Figure 3a) remained relatively stable over the study period. However, concentrations in the HH herd lowered from a median of 2.08 pg mg^-1^ across both winter timepoints to 1.26 and then 0.93 pg mg^-1^. This yielded a significant difference between herds across the time points (*F* = 6.87, *P* = 0.011). There was also a significant effect of time (*F =* 3.39, *P* = 0.020). Temporal variations in nasal mucus cortisol concentration (Figure 3b) were significant (*F* = 8.083, *P* < 0.001). However, the difference between herds was not significant across all time points (*F* = 2.37, *P* = 0.130). For the HH herd, concentrations increased from Winter 1 to Summer 1, with a sharp drop off in Summer 2. Whilst a similar shape is seen for the HG herd, it is less extreme and also earlier with a peak in Winter 2 and reduction through the Summer.

**Fig. 4:**
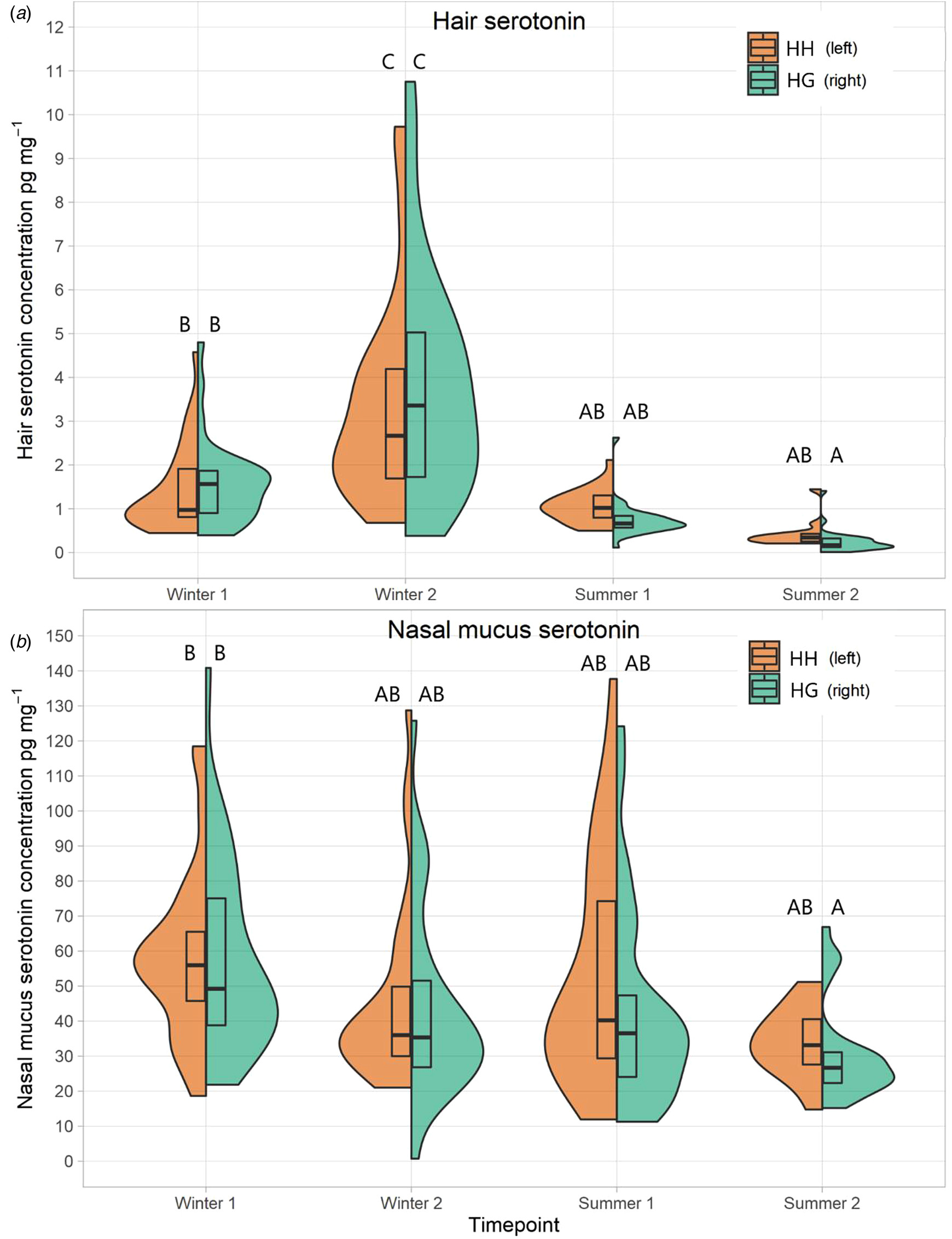
Split violin plots of serotonin concentrations (pg mg^-1^) for both herds across all time points in both hair (a) and nasal mucus (b). The left half of each plot is the HH herd (coloured in HH), and the right half is the HG herd (coloured in HG). Boxes in the middle represent Q1, median and Q3. Plots that do not share a letter are significantly different to one another.

Hair serotonin concentrations (Figure 4a) showed significant temporal variation across the study period (*F* = 56.51, *P* < 0.001). This was characterised by an increase in concentrations over winter followed by a sharp drop off in summer, for both herds. There was no significant difference in concentrations between herds (*F* = 0.019, *P* = 0.892). Concentrations of serotonin within nasal mucus (Figure 4b) varied significantly over time (*F* = 6.19, *P* < 0.001). Across both herds, there appeared to be a gradual decrease over winter which somewhat levelled out moving through the summer. Whilst nasal mucus serotonin concentrations were higher in the HH herd than HG, this was not significant at α = 0.05 (*F* = 6.19, *P* = 0.071).

### Animal performance

**Fig. 5:**
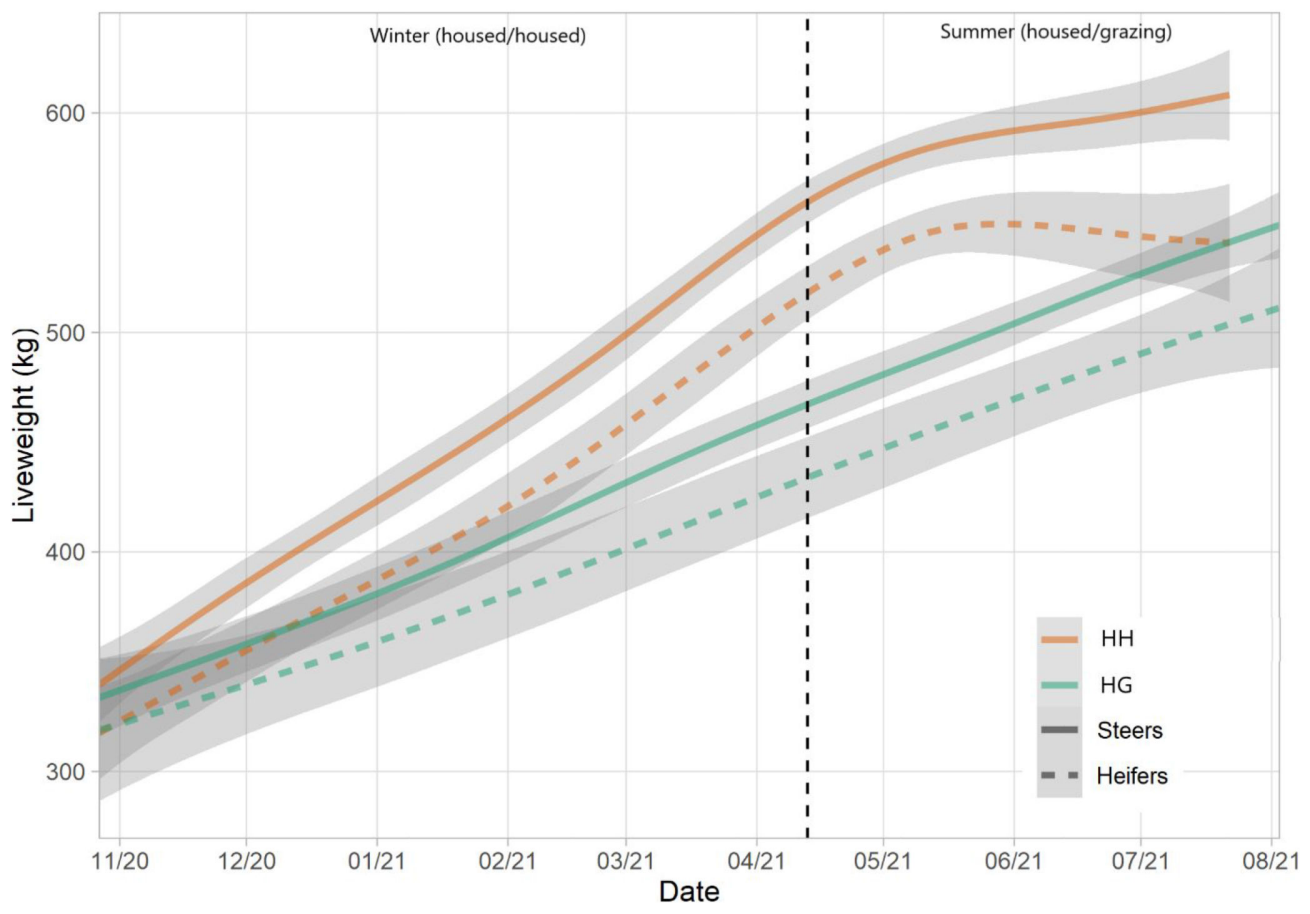
Liveweights of HH and HG herds over the study period, split by sex. Lines represent means and shading represents 95% confidence intervals.

Average daily gain (ADG) was significantly greater for the HH herd (1.21 kg day^-1^) compared to HG (0.74 kg day^-1^) (*t* = 13.95, *P* < 0.001) (Table 4 and Figure 5). A significant difference was also observed between breeds with ST cattle yielding a greater rate (1.01 kg day^-1^) than STX (0.83 kg day^-1^) (*t* = 3.11, *P* = 0.003). Despite steers finishing with a greater liveweights than heifers, they started the study period at a greater weight and thus differences in ADG were not significant (*t* = 1.58, *P* = 0.119).

### Metric correlations

**Fig. 6:**
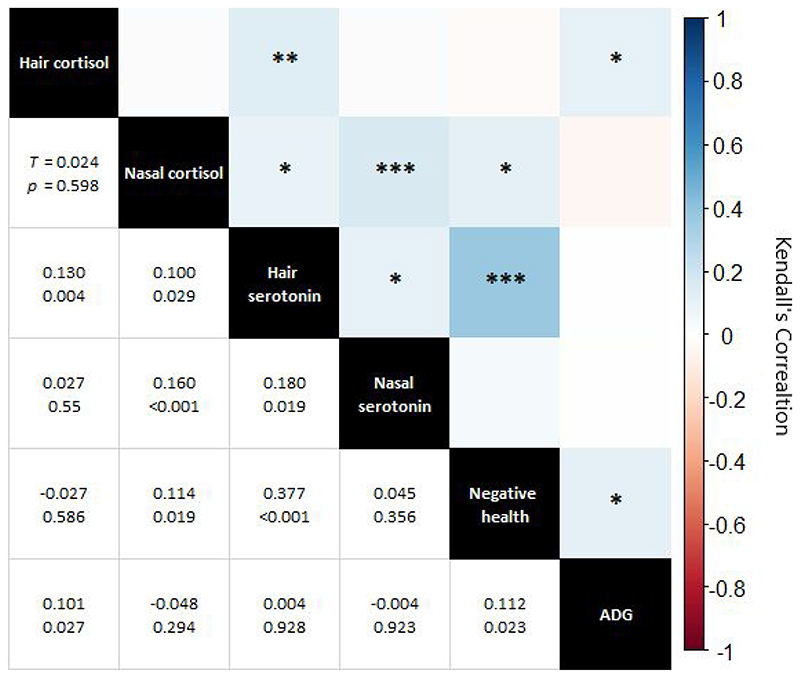
Correlation matrix of hormonal measures, negative health, and average daily gain (ADG). Top right of chart: visual representation of correlations with colour indicating Kendall’s Tau and asterixis representing p-value (0.050 * 0.010 ** 0.001 *** 0.000). Bottom left, specific Kendall’s Tau and p-value for each correlation pairing.

Metric correlations were statistically significant for 8 of 15 parings (Figure 6). There was a moderate and statistically significant (τ = 0.377, *P* < 0.001) correlation of hair serotonin with negative health. The other significant correlations were weak (τ = 0.180 or less).

## Discussion

Physical indicators of health were broadly similar across the two systems. Whilst there was a significantly higher incident rate of nasal discharge for HH, this was true from the start of the study period. It continued through winter before prevalence reduced over summer. Given that, nasal discharge prevalence cannot confidently be attributed to grazing/housing. Temporal trends were far stronger than inter-group trends, with negative trait scores being higher in late winter and improving over summer. Research by de Graaf *et al*. (2017) found that welfare indicators scored less favourably towards the end of housing periods, suggesting that prolonged housing may be a risk-factor to welfare. However, the fact that scores for HH improved over summer, despite remaining housed suggests that this phenomenon is a combined impact of housing and cold, wet, weather - not purely a consequence of housing itself. This interpretation is supported by Wagner *et al*. (2018), who found that the overall welfare of dairy cattle (assessed by the Welfare Quality protocol) improved from winter to summer. Considering nasal discharge as an example, it is broadly accepted that respiratory infections are most common in winter (Svensson *et al*., 2006), however, the increased proximity of cattle in housing is also a risk factor (Lago *et al*., 2006). Incidences of hairless patches were marginally more prevalent in cattle that were housed than grazing. This was attributed to potential cases of ringworm, which is more common in housed cattle due to darker, warmer, and more moist conditions and due to the proximity to potential sources of infection (Rook and Frain-Bell, 1954). Reflecting these limited differences in physiological health indicators, the extent of non-routine veterinary treatments was broadly similar between both herds. Whilst there were more treatments for eye conditions for the HG herd, this occurred during winter, when both were housed, and thus cannot be attributed to a difference in management. The mechanical chopping and spreading of straw for bedding deployed in the daily routine can create a lot of airborne particulates and this is a noted risk factor of infectious bovine keratoconjunctivitis. Consequently, staff are particularly vigilant in responding to the signs of this condition due to historic incidences. It is unlikely that the differences between the herds were due to underreporting (*personal communication*, 2021). Regarding the four incidences of mange in the HH herd, the condition may be associated to barn conditions (Sarre *et al*., 2012) (it is sometimes referred to as ‘barn itch’) and the increased ability for *Psoroptes ovis* to spread in barn conditions, compared to pasture (Sweatman, 1958).

The greater growth rate of the HH herd, compared to HG, was to be expected. The housed system was designed to be representative of real-world practice and thus was relatively intensive with the animals receiving rolled barley and distiller’s grain. Both herds grew at relatively steady rates suggesting no significant limiting factors (e.g., disease) to growth outside of diet. This was reflected in BCS data as scores for both herds increased over time towards finishing.

During winter, QBA outcomes of both herds were similar, reflecting their comparable housing. The dispersal was relatively broad across PC1 and PC2, similar to results by Cooke *et al*. (2022) that assessed a previous years cohort of cattle in the same facilities. The reason for the slightly greater variation of QBA in the HH herd, and inclination towards “Agitated”, “Playful”, and “Inquisitive” is unknown. It may be a consequence of the higher energy density of the diet and less energy spent in moving whilst eating, thus providing more time and energy for other activities. Summer QBA results showed a notable difference in the overall behaviour profiles of the two groups. The HG herd (grazing) were highly associated with descriptors such as “Content”, “Happy”, “Sociable”, and “Positively Occupied”. This may be a consequence of a high amount of time spent actively grazing as a cohesive herd unit. Anecdotally, HH animals appeared to value and utilise free space more and perform independent activities, compared to HG who had greater social cohesion. HH animals appeared to spend less time feeding which led to periods when they were awake and alert but were not “Positively Occupied” leading to them being “Bored”, often gazing vacantly. This outcome is not dissimilar to results from Krohn *et al*. (1992), who found greater behavioural synchronisation of dairy cattle on pasture than in housing. These results highlight the potential for boredom in indoor housing and consequently the potential benefits of environmental enrichment within cattle sheds (Mandel *et al*., 2016). The HH herd showed a greater variation in behaviour, this is possibly because they had to spend less time roaming and feeding, thus there was more time for various other activities. It may also be a consequence of the reduced herd size as summer progressed, which could have reduced the effect of social synchronicity, increased the relative weighting of each individual’s behaviour, and provided more space (lower stocking density) to express some behaviours. This difference in behaviour needs further investigation and testing cattle preference or developing a specific ethogram with this in mind could provide key insight. Free-choice experiments (as has been carried out in dairy cattle [Falk *et al*., 2012; Legrand *et al*., 2009]), giving cattle access to both high quality housing and pasture would perhaps be the most effective method to assess which system/environment best suits the psychological and emotional needs of beef cattle. When interpreting the PCA results from QBA, it is important to consider potential observer bias (Tuyttens *et al*., 2014) and in this study a single assessor was used,) though this is not uncommon (Muri *et al*., 2019; Temple *et al*., 2013) and the assessor had previously shown high inter-observer reliability with other assessors (Cooke *et al*., 2022) The extent of behavioural differences between herds were particularly strong and thus subtle subconscious bias to any meaningful extent seems improbable. On the whole, loadings for PC1 and PC2 were relatively weak (-0.442 to 0.423) with no particularly extreme values, possibly due to a level of multicollinearity of terms.

Within the HG herd, there was a rise of nasal mucus cortisol concentrations over winter, which appeared to drop off after turn-out and through summer. Whilst this could indicate a beneficial impact of turn-out and grazing, there was also a decrease for the HH herd over summer. Also, hair cortisol concentrations were lower in the HH herd compared to HG at both summer samplings. It may be that both herds are seeing different positives. For example, the HH herd had a lower stocking density compared to the stocking density and the time that their cortisol levels peaked, whilst the HG herd experienced natural grazing outdoors. Furthermore, during the summer period, compared to the HG herd, the HH herd had a relatively sedentary lifestyle with no need or opportunity to graze. They were also less exposed to weather events, notably cool nights, rain, and heat/sun, reducing potential risks of climatic stress, though at no point in the study period did the temperature humidity index exceed 72, which was noted by Higashiyama *et al*. (2013) as a threshold for an elevated cortisol response. Mean hair cortisol concentrations seen in the HG herd across both summer samplings (2.41 pg mg^-1^) was towards the lower ends of those observed in cattle elsewhere [12.15, (González-de-la-Vara *et al*., 2011), 5.7 (Burnett *et al*., 2014), 3.49 (Creutzinger *et al*., 2017), 2.5 (Comin *et al*., 2011), 2.35 (Moya *et al*., 2013)]. Cortisol levels are particularly responsive to acute forms of stress and thus may not be sensitive to the more subtle and long-term welfare implications of housing/grazing in herds of well-cared for cattle. Furthermore, although often referred to as the ‘stress hormone’, cortisol is more complex than that. It coordinates a variety of metabolic process and interacts with other hormones, opening up the possibility of non-stress related factors (e.g. diet) impacting concentrations (Thau *et al*., 2023).

Whilst serotonin concentrations changed over time, at any one timepoint they were broadly similar between both herds. Importantly, serotonin concentrations were not lower in the HH herd compared to the HG during the summer, suggesting that exposure to sunlight was not a controlling factor – though the extent to which cattle serotonin synthesis is impacted by sunlight is mostly unknown. The cause for the temporal pattern in serotonin concentrations in both hair and nasal mucus was not clear. However, the moderate positive correlation of hair serotonin with negative health may be driving this. Serotonin plays important roles in the immune process, including acting as a pro-inflammatory mediator and in regulating cytokine production (Wu *et al*., 2019) and thus might be elevated in response to infection, injury, or other health concerns. However, this same trend was not seen for nasal serotonin.

With regards to nasal mucus, it is possible that the composition of the mucus changed over time, perhaps in response to factors such as age, growth, weather and sub-clinical respiratory infection, which could limit the interpretation of nasal mucus results due to variability that may add both temporally and between individuals. For example, in humans, respiratory infections have been shown to influence the consistency of nasal mucus (Yuta *et al*., 1998), which may influence the relative concentrations of its constituents. The statistically significant, albeit weak (τ = 0.160), correlation between nasal cortisol and nasal serotonin supports this. Ultimately, the use of non-invasive endocrinological methods in animal welfare requires further development and testing.

The animals included in this study were managed to ‘best practice’ standards and were also covered by the Animal Scientific Procedures Act (1986). Consequently, the quality of care and resources available for husbandry were high for both herds and the interpretation of results is limited by that context. In other circumstances, risk factors associated with housing or grazing may be present or exacerbated in ways not evident from this study and the likelihood of this may not be equal across both systems. For example, less frequent ‘mucking out’ could increase disease risk during housing (Peeler *et al*., 2000) or turning cattle out onto inappropriate ground could increase risk of lameness (Tibbetts *et al*., 2006). The decision to house or graze cattle may also be associated with other management decisions that influence animal welfare. Certainly, many farm accreditation schemes (e.g., Soil Association organic status) require minimum grazing periods as part of a wider array of commitments. This study aimed to control for such variables to deliver a controlled study, the next step is to expand research to a wider number of farms to take into account and assess these potentially confounding/conflicting variables.

Results provide limited evidence that summer grazing is beneficial to cattle welfare. This is predominantly evidenced through behavioural indicators of welfare, with some support from physical indicators. Due to the behavioural data, this study was able to draw conclusions beyond those elsewhere, such as those reported by Wagner *et al*. (2018) who found that grazing time had no overall impact on the welfare of dairy cattle (as measured by the Welfare Quality protocol). However, evidence was not clear across all indicators of welfare, largely due to low incidence of negative health indicators, due to generally good levels of husbandry in both systems. This reinforces the need for holistic approaches to assessing welfare and whilst these have been presented elsewhere (e.g., Welfare Quality Protocol) few include molecular techniques, whether that be endocrinological, immunological, or other. Though endocrinological data from this study provides only limited evidence of cortisol and serotonin being useful indicators for routine/regular assessment. It suggests that the quality of a system’s management is also a significant driver of welfare than the exact system itself. Indeed, Park *et al*. (2020) identified a number of features (e.g. flooring type, stocking density) across different beef cattle housing systems and how they can positively and negatively impact animal welfare. Finally, significant temporal trends were present across all types of indicators, highlighting the potential risk of welfare losses during winter housing.

### Conclusion

This study found evidence that summer grazing periods are beneficial to beef cattle welfare, compared to housing. This conclusion was predominantly borne out through behavioural assessments, highlighting not only the role grazing has in livestock production, but also how welfare must be approached holistically using multiple types of measures. Strong temporal differences in welfare, across numerous indicators, highlight the potential for negative welfare outcomes during winter housing and thus housing conditions are a key area for intervention to improve cattle welfare. The study also highlights the need to develop and standardise holistic approaches to animal welfare assessments that are inclusive of molecular techniques.

## Supplementary Material

Supplementary Material

## Figures and Tables

**Table 1:** Top: List of physical indicators and description of scoring. “n/a” signifies that that value is not possible and thus the indicator is a binary measure. Bottom: body condition scoring, scores and relevant description (NADIS, 2010).

Physical indicator scores			
Indicator	0	1	2
Cleanliness	No dirt patches larger than a hand	Dirt patches equating to greater than a hand size but shorter than a forearm	Dirt patches of forearm size or greater
Diarrhoea	No diarrhoea	n/a	Signs of diarrhoea
Hairlessness	No hairless patches >2cm diameter	n/a	Hairless patches >2cm
Lameness	No lameness	Impaired mobility or uneven weight bearing, immediately identifiable	Severely impaired mobility, unable to meet normal walking pace
Lesions	No lesions >2cm	n/a	Lesions >2cm evident
Nasal discharge	No discharge >3cm	n/a	Discharge >3cm present
Ocular discharge	No discharge >3cm	n/a	Discharge >3cm present
Swelling	No swelling >2cm	Mild swelling such that normal anatomy of area is enlarged. Poorly defined.	Substantial abnormal swelling that is prominent away from the body
	Body condition scoring		
BCS	Description		
1. Poor	Notable cavity at tail base with no fatty tissue. Spine and ribs prominent and sharp horizontal process. Skin potentially rough.		
2. Moderate	Shallow cavity and tail base, but prominent pin bones and some fat under skin. Horizontal process visible. Skin typically supple.		
3. Good	Some depression noticeable in the loin with horizontal process not clearly visible, but able to be felt. Fat cover around base of tail. Pelvis can be felt.		
4. Fat	Tail head area has full fat cover. Areas of excess fat are evident. Bones cannot be felt around the loin and appears visually rounded.		
5. Grossly fat	Tail head is partially buried/depressed in fatty tissue. Bones around the loin cannot be felt, irrespective of pressure applied, appears rounded and plump.		

**Table 2:** Summary of non-routine veterinary treatments divided by season, herd, and condition. The number refers to the total number of treatments administered across all animals, the number in brackets refers to the total number of animals treated. A hyphen (-) signifies no treatment.

	Number of treatments (number of animals)			
	Winter		Summer	
	HH	HG	HH	HG
Eye condition	2 (1)	11 (4)	-	-
Worms	3 (3)	7 (7)	-	-
Bloat	-	-	4 (4)	-
Mange/lice	-	-	-	1 (1)
Skin condition	-	-	-	1 (1)
Tail injury	-	-	-	3 (1)

**Table 3:** Loadings of each term for PC1 and PC2. Terms are ranked based on highest to lowest value.

PC1: Arousal		PC2: Mood	
Term	Value	Term	Value
Calm	0.351	Pos. occ.	0.423
Relaxed	0.348	Active	0.384
Content	0.333	Happy	0.374
Happy	0.260	Sociable	0.339
Pos. occ.	0.205	Lively	0.234
Sociable	0.163	Friendly	0.216
Friendly	0.079	Content	0.188
Indifferent	0.049	Irritable	0.117
Bored	0.105	Agitated	0.108
Active	-0.064	Uneasy	0.078
Playful	-0.147	Playful	0.071
Inquisitive	-0.235	Inquisitive	0.013
Irritable	-0.282	Indifferent	-0.074
Uneasy	-0.289	Relaxed	-0.081
Lively	-0.323	Calm	-0.198
Agitated	-0.389	Bored	-0.442

**Table 4:** Summary information of cattle performance in relation to slaughter.

	HH	HG
ADG: weaning to slaughter (kg)	1.2 (0.19)	1.0 (0.10)
ADG: birth to slaughter (kg)	1.2 (0.12)	1.1 (0.09)
Slaughter LW (kg)	602 (34.8)	609 (35.4)
Slaughter age (days)	462 (35.4)	545 (56.0)
