# Supplementary material for: Comparison of the welfare of beef cattle in housed and grazing systems: hormones, health, and behaviour
Source: J Agric Sci. Author manuscript; Available in PMC 2023 Aug 28. (PMC7614983; doi:10.1017/S0021859623000357)
Supplement: Supplementary Material [file EMS178536-supplement-Supplementary_Material.pdf]

Comparison of the welfare of beef cattle in housed and grazing systems: hormones, health, and behaviour

Cooke, A. S.[1,2], Mullan, S.[3,4], Morten, C.[1], Hockenhull, J.[4], Le Grice, P.[1], Le Cocq, K.[1,5], Lee, M.R.F.[1,4,5], Cardenas, L.M.[1], and Rivero, M.J.[1]

1. Net Zero and Resilient Farming, Rothamsted Research, Okehampton, UK
2. School of Life Sciences, College of Science, University of Lincoln, Lincoln, UK
3. UCD School of Veterinary Medicine, University College Dublin, Dublin, Ireland
4. Bristol Veterinary School, University of Bristol, Bristol, UK
5. School of Sustainable Food and Farming, Harper Adams University, Edgmond, UK

Supplement A – Weather patterns

Ambient weather (temperature, relative humidity, wind speed, solar radiation) was recorded at 15 min intervals by a weather station located approximately 1.6 km SE of the barns, adjacent to the fields grazed by the HG herd (Figure A). Temperature and relative humidity were recorded within the HH herd's barn. For both ambient and barn conditions, the adjusted temperature humidity index (THI) was calculated (Equation A) (Mader et al., 2006).

Equation A - Formula for temperature humidity index (THI) where: T = air temperature (°C), RH = relative humidity (%)

$$\text{THI} = 0.8T + 0.01RH(T - 14.4) + 46.4$$

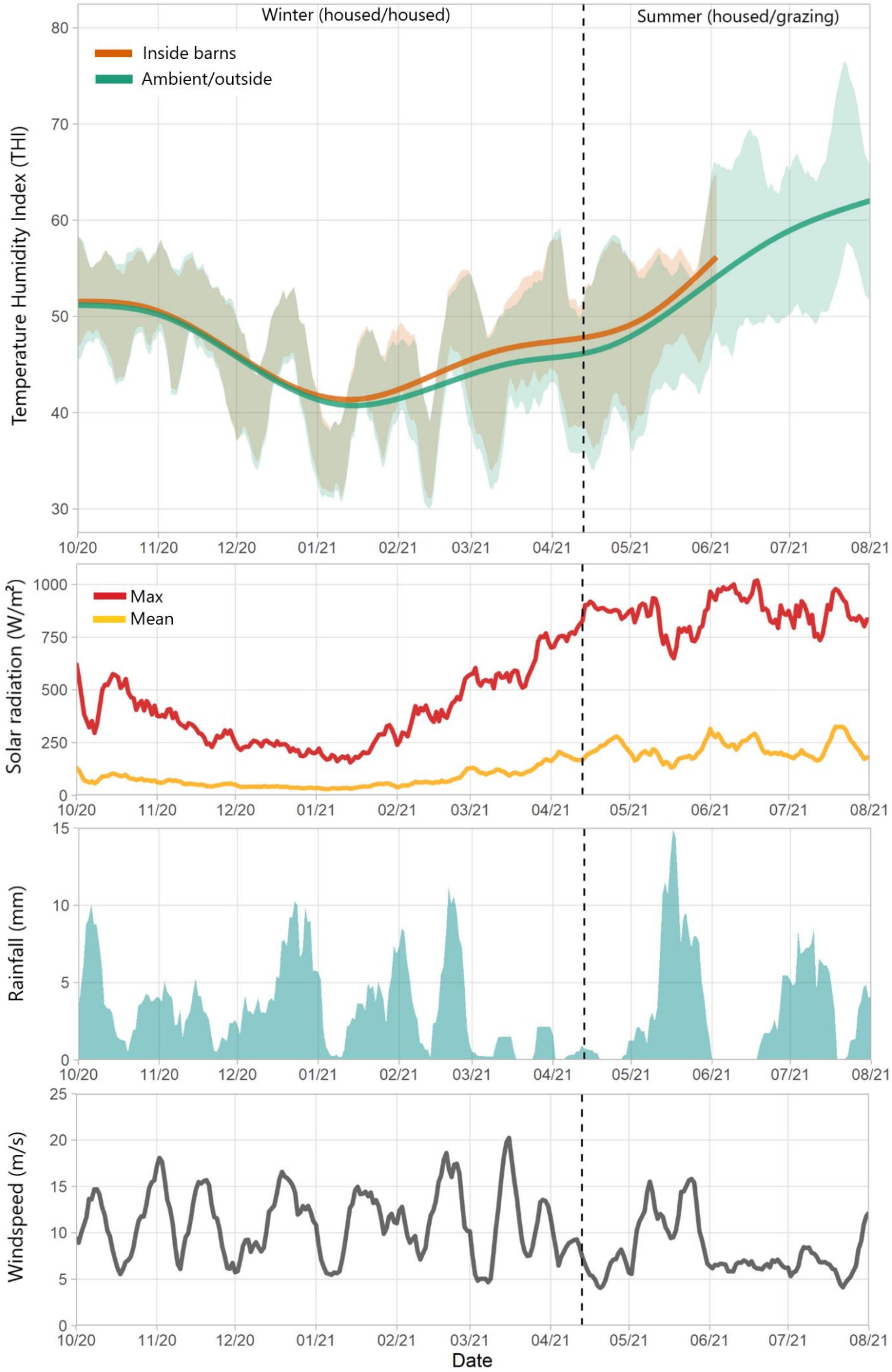
Winter (housed/housed)
Summer (housed/grazing)
Inside barns
Ambient/outside
Temperature Humidity Index (THI)
80
70
60
50
40
30
10/20
11/20
12/20
01/21
02/21
03/21
04/21
05/21
06/21
07/21
08/21
Solar radiation (W/m²)
1000
750
500
250
0
Max
Mean
Rainfall (mm)
15
10
5
0
Windspeed (m/s)
25
20
15
10
5
0
Date

Figure A - Weather data covering the experimental period. The vertical dashed line represents turnout date for the HG herd. **Top**: Temperature Humidity Index (THI) scores for both inside (HH line, higher) and outside (HG line, lower) the barns. Lines represent daily means with a generalized additive model (GAM) applied. Shaded areas represent a 7-day rolling average of maximum and minimum daily THI. **Top-middle**: Solar radiation (W/m^2). The line (top) represents a 7-day rolling average of maximum solar radiation whilst the orange line (bottom) represents a 7-day rolling average of mean solar radiation. **Bottom-middle**: Total rainfall per day (mm) on a 7-day rolling mean. **Bottom**: Windspeed (m/s) on a 7-day rolling mean.

Comparison of the welfare of beef cattle in housed and grazing systems: hormones, health, and behaviour

Cooke, A. S.[1,2], Mullan, S.[3,4], Morten, C.[1], Hockenhull, J.[4], Le Grice, P.[1], Le Cocq, K.[1,5], Lee, M.R.F.[1,4,5], Cardenas, L.M.[1], and Rivero, M.J.[1]

1. Net Zero and Resilient Farming, Rothamsted Research, Okehampton, UK
2. School of Life Sciences, College of Science, University of Lincoln, Lincoln, UK
3. UCD School of Veterinary Medicine, University College Dublin, Dublin, Ireland
4. Bristol Veterinary School, University of Bristol, Bristol, UK
5. School of Sustainable Food and Farming, Harper Adams University, Edgmond, UK

Supplement B – Herd size

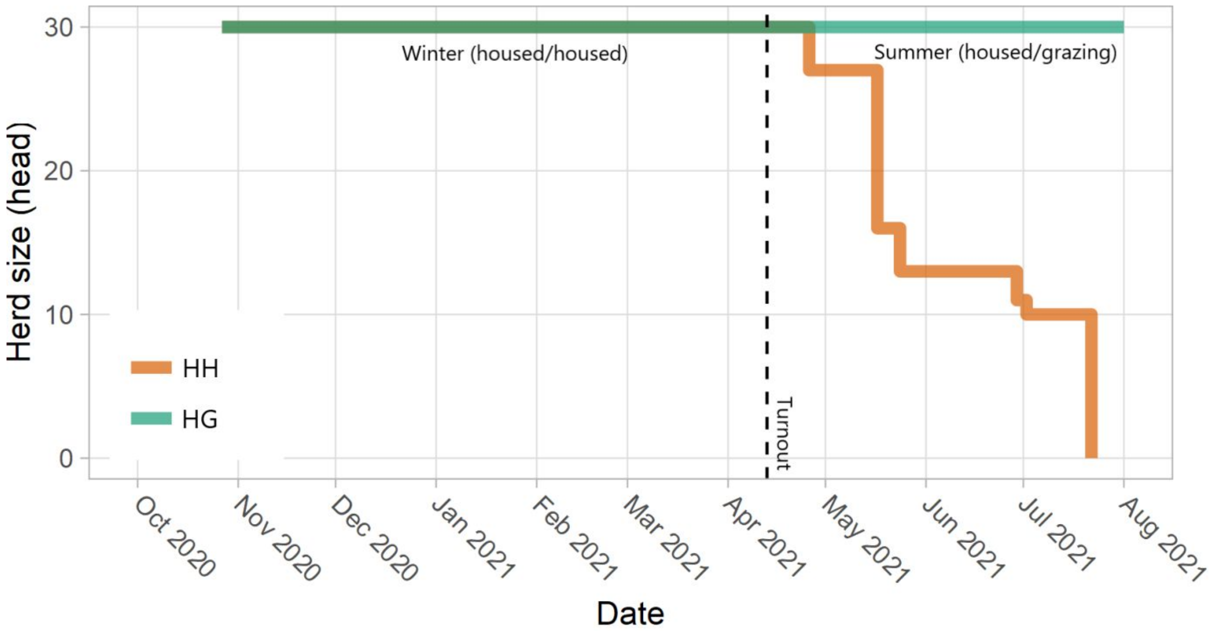

Figure B - Head count of each herd over the course of the study period.

Comparison of the welfare of beef cattle in housed and grazing systems: hormones, health, and behaviour

Cooke, A. S.[1,2], Mullan, S.[3,4], Morten, C.[1], Hockenhull, J.[4], Le Grice, P.[1], Le Cocq, K.[1,5], Lee, M.R.F.[1,4,5], Cardenas, L.M.[1], and Rivero, M.J.[1]

1. Net Zero and Resilient Farming, Rothamsted Research, Okehampton, UK
2. School of Life Sciences, College of Science, University of Lincoln, Lincoln, UK
3. UCD School of Veterinary Medicine, University College Dublin, Dublin, Ireland
4. Bristol Veterinary School, University of Bristol, Bristol, UK
5. School of Sustainable Food and Farming, Harper Adams University, Edgmond, UK

Supplement C – Description of QBA terms

The following are brief practical definitions of the terms used within the study, written for the purpose of aiding assessment in the field.

ACTIVE – The state of doing something that requires movement, even if that is just chewing or grooming. The more continuous and pronounced the movement, the more active.

AGITATED –Behaviours of any animals being altered due to non-self-factors (e.g. other cattle, human presence).

APATHETIC – More severe than indifferent, animals ignoring / not responding to things that you would expect them to typically react to and/or not noticing stimuli they would be expected to.

BORED – A lack of being positively occupied (though not necessarily negatively occupied) and/or lack of positive stimuli.

CALM – An absence of stressors, fear, and frantic or irrational behaviour.

CONTENT – A state in which the animals are free from stressors and able to perform the behaviours they want to at their own leisure without fear or inhibition.

DISTRESSED – The most 'powerful' term on this list. Distress is an extreme form of unease, manifested in behaviours that may be panicked and irrational. For example, violent bucking, trying to force their way out of pens, highly aggressive non-play fighting

FEARFUL – Applies to fear towards any stimuli (animals, humans, farm vehicles etc) in the immediate environment.

FRIENDLY – Direct positive interaction with other animals. For example, grooming, playing, sharing food.

FRUSTRATED – Struggling or being unable to do something they desire to. For example, if an animal blocked their access to a resource or if feed was out of reach.

HAPPY – More positive than 'content' and calm (e.g. the absence of stressors) – the derivation of pleasure received from the environment and behaviour and the presence and reception of positive stimuli.

INDIFFERENT (negative) – Animals not actively responding to variations in their environment. They may notice and watch, but not react (e.g. get up and move).

INQUISITIVE – The investigation of stimuli and objects (inc. live objects) in their environment.

IRRITABLE – Sensitivity and annoyance by potential stressors (e.g. other animals, their environment disrupting them).

LIVELY – This is stronger than active, all lively animals are active, but not all active animals are lively. The strongest signs of liveliness are actions such as trotting, running, jumping/bucking.

PLAYFUL – Positive interaction with objects or animals for enjoyment purposes.

POSITVELY OCCUPIED – Performing behaviours which are positive to their welfare and general maintenance (e.g. eating, sleeping, drinking).

RELAXED – Feeling comfortable and safe, with minimal stress and unwanted exertion.

SOCIABLE – This is more general than friendly. One example might be if animals chose to feed next to each other, opposed to being spread out, or if they all sleep close together. It is also the synchronicity and cohesion of behaviour.

UNEASY – Animals seeming to be nervous and not calm. For example, if they are stopping behaviour to keep watch on something or if they are jumpy/sensitive to noise and movement.

Comparison of the welfare of beef cattle in housed and grazing systems: hormones, health, and behaviour

Cooke, A. S.[1,2], Mullan, S.[3,4], Morten, C.[1], Hockenhull, J.[4], Le Grice, P.[1], Le Cocq, K.[1,5], Lee, M.R.F.[1,4,5], Cardenas, L.M.[1], and Rivero, M.J.[1]

1. Net Zero and Resilient Farming, Rothamsted Research, Okehampton, UK
2. School of Life Sciences, College of Science, University of Lincoln, Lincoln, UK
3. UCD School of Veterinary Medicine, University College Dublin, Dublin, Ireland
4. Bristol Veterinary School, University of Bristol, Bristol, UK
5. School of Sustainable Food and Farming, Harper Adams University, Edgmond, UK

Supplement D – Hair and nasal mucus preparation

Hair samples were taken at the same time as physical checks were conducted. Hair was taken using an electric shearer, in the area around the base of the neck and between shoulder blades and stored at -20ºC prior to further preparation. Approximately 250 mg of hair was placed in a beaker to be washed four times. For the first two washes: 5 ml of water was added to the beaker which was then shaken at 100 rpm for 3 mins, after which the water was strained off. The third and fourth wash followed a similar process but with isopropanol in place of water. After the final wash samples were dried at 30ºC for 3 days. Hair samples were then ground in a ball mill at 50 hz for 2 mins until a powder of approximately 2 mm. A 50 mg sub-sample of the resulting ground hair was then weighed into a 2 ml microcentrifuge tube and 1.5 ml of methanol added. Tubes were vortexed for 10 secs, then sonicated for 30 minutes, and then placed in an incubator-shaker at 100 rpm and 30ºC for 18 h. Samples were then centrifuged at 7,000 x g for 2 mins, after which 750 µl of the supernatant was transferred to a new 2 ml microcentrifuge tube. Tubes were then placed in a block heater at 38ºC for 18hrs to evaporate off the methanol from the supernatant. The sample was then resuspended by adding 150 µl of PBS and vortexing for 30 secs. Samples were then stored -20ºC until analysis.

Nasal mucus was collected using sterile swabs (Sterilin F155CA). Whilst animals were appropriately restrained the swab was inserted into the nostril at a depth of approximately 5 cm (not so far enough to feel resistance) and rotated around the inside of it. Samples were then stored -20ºC until analysis. Microcentrifuge tubes (2 ml) were weighed and a 45 µm filter then added to the tube. Swab tips were cut off and placed in the filter basket, 500 µl of methanol was added. Samples were then placed in a shaker at 100 rpm for 18 h at 20°C. Tubes were

centrifuged at 10,000 x g for 2 minutes to draw the methanol extract through the filter. The filter was then removed, and tubes placed in a block heater at 30ºC for 18h, to evaporate off the methanol. Tubes were re-weighed and the addition in weight considered to be equal to the mass of material extracted from the swab. The extracted material was re-suspended by adding 150 µl of PBS and vortexing for 30 seconds.

Comparison of the welfare of beef cattle in housed and grazing systems: hormones, health, and behaviour

Cooke, A. S.[1,2], Mullan, S.[3,4], Morten, C.[1], Hockenhull, J.[4], Le Grice, P.[1], Le Cocq, K.[1,5], Lee, M.R.F.[1,4,5], Cardenas, L.M.[1], and Rivero, M.J.[1]

1. Net Zero and Resilient Farming, Rothamsted Research, Okehampton, UK
2. School of Life Sciences, College of Science, University of Lincoln, Lincoln, UK
3. UCD School of Veterinary Medicine, University College Dublin, Dublin, Ireland
4. Bristol Veterinary School, University of Bristol, Bristol, UK
5. School of Sustainable Food and Farming, Harper Adams University, Edgmond, UK

Supplement E –Assay protocols

1.1 Cortisol assay

Cortisol assays were conducted by competitive ELISA using the Expanded Range High Sensitivity Salivary Cortisol Enzyme Immunoassay Kit, produced by Salimetrics (USA) and further details can be found on their website. In breif:

1. Reagents were brought up to room temperature
2. 24 ml of assay diluent was measured into a sterile tube.
3. 25 µl of each of six standards (3.0, 1.0, 0.333, 0.111, 0.037, 0.012 µg dl^{-1}) was pipetted into wells. 25 µl of blanks (assay diluent) and samples (no dilution) were added to their wells.
4. The enzyme conjugate was diluted (1:1600) into the assay diluent. 200 µl of this solution was added to each well.
5. The plate was placed on a shaker for 5 mins at 100 rpm, it was then incubated at 20°C for 1 hr.
6. Well liquid content was discarded, and the plate was washed four times using the buffer solution (diluted to 1x). For each wash, 300 µl of wash buffer was added to each well before being discarded. The plate was then blotted onto clean paper towels after each discard.
7. 200 µl of TMB substrate solution was added to each well.
8. The plate was placed on a shaker for 5 mins at 100 rpm before being incubated, in the dark, at 20°C for 25 mins.

9. 50 µl of stop solution was added to each well
10. The plate was placed on a shaker for 3 mins at 100 rpm.
11. The plate was scanned at 450 nm using a plate reader (Infinite 200 Pro, Tecan Life Sciences, Switzerland).
12. The optical densities of standards were used to create a four parameter logistic curve from which sample from which sample concentrations were interpolated (MyAssays Ltd.).

1.2 Serotonin assay

Serotonin ELISAs were conducted using a Serotonin High Sensitivity ELISA kit produced by DLD-Diagnostika GMBH (Germany) (product no. EA 630/96). Additional details and a full protocol can be found via their website, in breif:

1. Reagents were prepped in accordance to manufacturer instructions.
2. 20 µl of samples were pipetted into their respective wells.
3. 25 µl of acylation buffer was added.
4. 10 µl of acylation reagent was added.
5. The plate was incubated at room temperature for 1 hr.
6. 25 µl of deactivator solution was added.
7. The plate was covered and incubated at room temperature for 3 hrs.
8. 50 µl was taken from each well and added to a coated microtiter wells.
9. The plate was covered and incubated at 5°C for 18 hrs.
10. The plate was washed 4x with 300 µl wash buffer.
11. 100 µl of enzyme conjugate was added to all wells.
12. The plate was incubated at room temperature on an orbital shaker at 100rpm, for 60 mins.
13. The plate was washed 4x with 300 µul wash buffer.
14. 100 µl of substrate was added to all wells.
15. The plate was incubated at room temperature on an orbital shaker at 100rpm, for 30 mins.
16. 100 µl of stop solution was added to all wells.
17. The plate was scanned at 450 nm using a plate reader (Infinite 200 Pro, Tecan Life Sciences, Switzerland).

18. The optical densities of standards were used to create a four parameter logistic curve from which sample from which sample concentrations were interpolated (MyAssays Ltd.).
